# A New Hexaploid Species of *Isoetes* (Isoetaceae) From Fujian, China, Based on Morphological and Molecular Evidence

**DOI:** 10.1002/ece3.72140

**Published:** 2025-09-12

**Authors:** Moran Wang, Hui Shang, Wen Shao, Binjie Ge, Yigang Song, Yuehong Yan, Weimin Ma, Yufeng Gu, Hui Shen

**Affiliations:** ^1^ College of Life Sciences Shanghai Normal University Shanghai China; ^2^ Eastern China Conservation Centre for Wild Endangered Plant Resources Shanghai Chenshan Botanical Garden Shanghai China; ^3^ CAS Center for Excellence in Molecular Plant Sciences, Institute of Plant Physiology and Ecology Chinese Academy of Sciences Shanghai China; ^4^ The National Orchid Conservation & Research Center of Shenzhen Shenzhen Guangdong China

**Keywords:** aquatic plant, morphology, population genetics, quillwort, taxonomy, whole genome resequencing

## Abstract

A new endemic species of *Isoetes* from Fujian, China, which was previously identified as *I. orientalis* , is described and illustrated. In this study, we observed its leaf cross‐cut characteristics under a microscope and spore morphology characteristics under a scanning electron microscope, counted its chromosome number, and compared population genetic differences between this hexaploid and *I. orientalis* based on single nucleotide polymorphisms (SNPs) extracted from whole‐genome resequencing data. We found that this hexaploid differs from *I. orientalis* in both leaf morphology and spore ornamentation, and that the two also exhibit significant differences in population genetics. Based on this comprehensive evidence, we identified the hexaploid as a new species and named it *I. fokiensis*. Here, we formally describe this hexaploid species and provide an identification key to the *Isoetes* species reported from China.

## Graphical abstract

A new endemic species of *Isoetes* from Fujian, China, which was previously identified as *I. orientalis*, is described and illustrated. In this study, we observed its leaf across‐cut characteristics under the microscope and spore morphology characteristics under the scanning electron microscope, counted its chromosome number, and compared population genetic differences between this hexaploid and *I. orientalis* based on single nucleotide polymorphisms (SNPs) extracted from whole‐genome resequencing data. We found that this hexaploid differs from *I. orientalis* in both leaf morphology and spore ornamentation, and that the two also exhibit significant differences in population genetics. Based on this comprehensive evidence, we identified the hexaploid as a new species and named it *I. fokiensis*. Here, we formally describe this hexaploid species and provide an identification key to the *Isoetes* species reported from China.

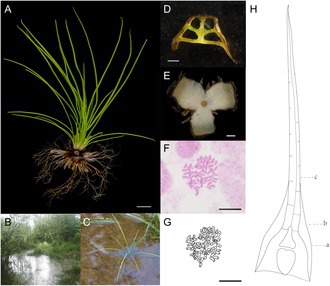

## 1 Introduction

Isoetaceae, often referred to as living fossils, are considered to have originated during the Late Devonian (359–383 Ma) (Pigg 1992; Kenrick and Crane 1997). *Isoetes* Linnaeus (1753: 1100), the sole extant genus within Isoetaceae, is widely distributed and comprises over 200 species worldwide (PPG I 2016; Troia et al. 2016). Up to now, 13 species of *Isoetes* have been reported in China (Handel‐Mazzetti 1923; Palmer 1927; DeVol 1972; Wang et al. 2002; Li et al. 2013; Liu et al. 2005, 2024; Li et al. 2019; Lu et al. 2021; Shu et al. 2022; Gu, Shu, et al. 2023; Tong et al. 2024). All species of this genus are listed in the IUCN Red List of Threatened Species (IUCN 2024) and are designated as National First‐Grade Protected Plants in China. According to the distribution of this genus over the world and the number of species in other countries with similar geography and climate to our country, we believe that there should be more undescribed species of quillwort in China.

*Isoetes* can be submerged aquatic, aquatic, or completely terrestrial, so we can find them at the bottom or edge of lakes, in wetlands, marshes, or agricultural fields (Troia et al. 2016). In addition to having a relatively stable ecological environment, all the species in this genus have similar and simple leaves with sporangia at the base (Hickey 1986; Pfeiffer 1922; Troia et al. 2016; Zhang and Taylor 2013). Therefore, it is difficult to delimit species just according to the leaf morphological characteristics, which has caused many cryptic species to exist in this genus (Gu, Xiang, et al. 2023). Spore ornamentation characteristics (Pfeiffer 1922; Hickey 1986; Takamiya 1999; Reed 1965) and chromosome number (Taylor and Hickey 1992; Dai et al. 2020; Hoot et al. 2004; Gu, Xiang, et al. 2023) provide stable evidence in identifying species, which are still considered significant features even now that molecular biology is prevalent.

Phylogenetic evidence has led to the recognition of new taxa within *Isoetes* (Moraolivo et al. 2016; Pereira et al. 2016, 2017, 2019; Li et al. 2019; Lu et al. 2021; Schafran et al. 2016). As researchers worldwide continue to uncover previously overlooked species, the systematics of the family has been reexamined (Schafran et al. 2018; Pereira et al. 2019, 2021; Choi et al. 2019; Dai et al. 2020; Singh et al. 2021; Suissa et al. 2022). To better understand *Isoetes* diversity in China, Gu, Xiang, et al. (2023) conducted a systematic phylogenetic and evolutionary study using complete chloroplast genome (plastome) data from nearly all Chinese *Isoetes* populations. Their analysis clearly separated two different populations of *I. orientalis* into distinct clades, which were assigned to the *I. sinensis* complex clade recovered in Dai et al. (2020) as the only hexaploid lineages within this group. However, the separation was supported by short branch lengths, indicating relatively small genetic divergence, possibly due to the limited sequence length and low number of variable sites in the chloroplast genome. In contrast, the nuclear genome harbours a far greater number of single nucleotide polymorphisms (SNPs) loci, providing higher resolution for distinguishing closely related lineages (Fu et al. 2022). To further clarify the relationship between the two populations, we therefore conducted population genetic analyses based on SNPs obtained from whole‐genome resequencing data.

In China, the haxaploid species *I. orientalis* was first found in Songyang County (Zhejiang Province), which was identified as *I. sinensis* for a long time for lacking further study (Liu et al. 2005). Another population of this haxaploid, distributing in Taining County (Fujian Province), was also identified as *I. orientalis*. To get a clear relationship between these two haxaploid populations assigned to the *I. sinensis* complex clade, besides spore morphology and chromosome counting, we also conducted supplementary population genetic analyses based on SNPs derived from whole‐genome resequencing data. At last, we determined the population living in Fujian Province as a new species instead of *I. orientalis* .

To clarify the relationship between these two hexaploid populations, we conducted supplementary population genetic analyses based on SNPs derived from whole‐genome resequencing data, in addition to spore morphology and chromosome counting. Ultimately, we determined that the population living in Fujian Province represents a new species rather than *I. orientalis* .

## 2 Materials and Methods

### 2.1 Taxon Sampling

Five living individuals were collected in June 2023 from two populations, Songyang County, Zhejiang Province (type locality), and Taining County, Fujian Province. These individuals were cultivated in the greenhouse of Shanghai Chenshan Botanical Garden. The outgroup *Isoetes changleensis* Yu C. Chen & Xing Liu is from the Changle Forest farm in Zhejiang Province (Table 1).

**TABLE 1:** Information of samples in this study.

Taxon code	Collection site	Population
SY08	Songyang, Zhejiang	SY
SY09	Songyang, Zhejiang	SY
SY12	Songyang, Zhejiang	SY
SY27	Songyang, Zhejiang	SY
SY28	Songyang, Zhejiang	SY
TN02	Taining, Fujian	TN
TN03	Taining, Fujian	TN
TN04	Taining, Fujian	TN
TN05	Taining, Fujian	TN
TN06	Taining, Fujian	TN
CL01	Changle Forest farm, Zhejiang	CL

### 2.2 Leaf and Sporangium Anatomy

Fully matured leaves of two populations were carefully collected, and their morphological features were examined. For measurement purposes, the leaf base (the attachment point to the rhizome) was designated as the starting point, and the leaf tip as the endpoint. The sporangial region of the leaf was imaged using a computer‐connected dissecting microscope (Nikon SMZ‐1500; Leica DFC450 C). For each sample, three sporangia were photographed. The length and width of the sporangia were measured using Adobe Photoshop CS5 software, and the average values were used to represent sporangium size. The sporangium characteristics were described following the definitions of leaf shape provided in *Plant Biology* (Zhou 2004). The rhizome of this new taxon was crosscut in the middle so that we can get a clear view of its shape.

### 2.3 Spore Morphology

Observation and measurement of spore morphology were carried out under a scanning electron microscope following the methodology of Gu (2022) and Gu, Xiang, et al. (2023).

The spore patterns of the samples were described according to published studies, including the 12 characteristics of the *Isoetes* spore pattern summarised by Hickey (1986), the spore pattern of ferns summarised by Ranker (1993) and the spore characteristics of Chinese *Isoetes* species described by Liu et al. (2008) and Gu, Xiang, et al. (2023). The ornamentation of the microspores was described based on the studies by Reed (1965).

### 2.4 Ploidy Determination

Chromosomes were counted using the methodology of Gu, Xiang, et al. (2023) and Zhang and Taylor (2013). Chromosome counting was performed using Photoshop CS5.

### 2.5 DNA Extraction, Whole‐Genome Resequencing and SNP Calling

Total genomic DNA was extracted from silica‐dried leaf tissues using the Tiangen Plant Genomic DNA Kit following the manufacturer's instructions. Whole‐genome resequencing was conducted at the China National GeneBank (CNGB) using the DNBSEQ platform with a paired‐end 150 bp (PE150) sequencing strategy.

The reference genome used for read alignment was based on the diploid *Isoetes taiwanensis* De Vol (Taiwania 17: 2, 1972), obtained from the National Center for Biotechnology Information (NCBI). To improve SNP calling specificity, we extracted the coding sequence (CDS) regions from the reference genome and used them as the mapping reference. CDS extraction was performed using gffread (https://github.com/gpertea/gffread), based on the GFF annotation file and a corrected FASTA file in which sequence headers were edited to match annotation identifiers. Both the GFF and corresponding FASTA files were downloaded from CoGe (Genome ID: 61511) (Wickell et al. 2021). The resulting CDS FASTA file was used as the reference for SNP calling.

Raw sequencing reads were first assessed and filtered using FastQC v0.20.0 (Chen et al. 2018) to remove adapter sequences and low‐quality bases (Andrews 2020). Clean reads were aligned to the CDS reference using BWA‐MEM v0.7.18 (Li and Durbin 2009), and the resulting BAM files were converted and sorted using SAMtools v1.20 (Li et al. 2009). PCR duplicates were marked and removed using GATK v4.1.9.0 (McKenna et al. 2010). Variant calling was carried out using the HaplotypeCaller and GenotypeGVCFs modules in GATK (Van der Auwera and O'Connor 2020). Only high‐quality biallelic SNPs and monomorphic sites were retained, with a minimum Phred quality score > 30. SNPs failing GATK hard filtering criteria (QD < 2.0 || FS > 60.0 || MQ < 40.0 || QUAL < 30.0 || MQRankSum < −12.5 || ReadPosRankSum < −8.0 || SOR > 3.0) were excluded. Additionally, sites with extremely low or high coverage (mean depth < 15 or > 300) were filtered out. The resulting SNPs were compiled into VCF format. For downstream analyses, a separate VCF dataset excluding the outgroup *I. changleensis* was also generated.

### 2.6 Phylogenetic Analysis and Population Genetic Diversity

SNPs were first quality‐filtered using PLINK version 1.9 by removing loci with a minor allele frequency (MAF) below 0.05, genotyping rate below 95%, or significant deviation from Hardy–Weinberg equilibrium (*p* < 0.0001) (Wills 2007). The filtered dataset was then converted into binary PED format and used for subsequent analyses. For genetic structure inference, ADMIXTURE version 1.3.0 was employed to estimate ancestry proportions across *K* = 1–5 clusters, with the optimal *K* determined by the lowest cross‐validation (CV) error. CV errors for each *K* value were extracted and visualized in R using ggplot2. Cluster membership outputs (QC.K.Q files) were manually matched with sample IDs and visualized using TBtools version 2.322.

For principal component analysis (PCA), the same filtered SNP dataset was input into GCTA version 1.94.1 to generate a genetic relationship matrix (GRM), followed by eigen decomposition to compute the first 10 principal components. The resulting eigenvectors were plotted using ggplot2 in R to visualize population clustering.

Maximum likelihood (ML) analyses were conducted using IQ‐TREE v1.6.12 (Nguyen et al. 2015) based on the CDS‐region SNP dataset of 11 *Isoetes* individuals, including an outgroup. The analysis was performed with 10,000 bootstrap replicates, and the best‐fitting model was selected using ModelFinder (Kalyaanamoorthy et al. 2017) and implemented in IQ‐TREE.

## 3 Results and Discussion

### 3.1 Morphology

**FIGURE 1:**
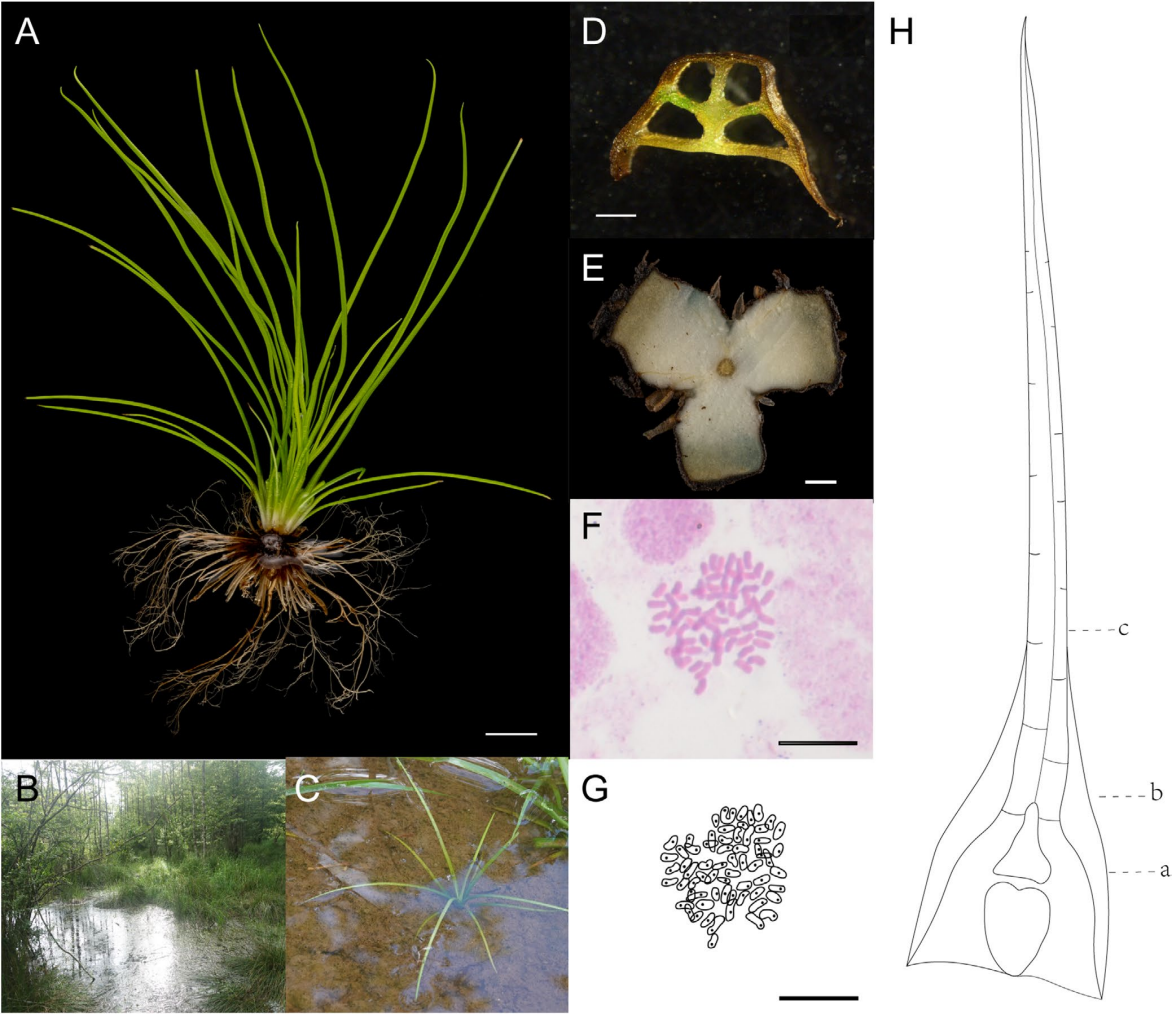
*Isoetes fokiensis* Y.F.Gu, M.R. Wang & Y.H.Yan, (A) Plants. (B) Habitat. (C) *I. fokiensis* in situ. (D) Transection at the lower part of the leaf, four distinct lacunae and a prominent leaf wing are present (Hb). (E) Transection of rhizome (3‐lobed). (F) Chromosomes in a single cell. (G) The lined drawing of chromosomes. Scale bars: D = 200 μm; A and E = 2000 μm; F and G = 10 μm. (H) Line drawings showing transverse sections at three sampling positions: (a) leaf base, just above the sporangium; (b) lower part of the leaf, further away from the sporangium; (c) middle part of the leaf, where the leaf wing begins to disappear.

*Isoetes fokiensis* is about 20–30 cm tall (Figure 1A) and grows in shallow mudflats (Figure 1B) as a perennial semi‐submerged plant (Figure 1C). The base of the leaf is trapezoidal in cross‐section (Figure 1D). The transection of the rhizome presents a 3‐lobed type (Figure 1E). Chromosome number is 66 (Figure 1F,G).

**FIGURE 2:**
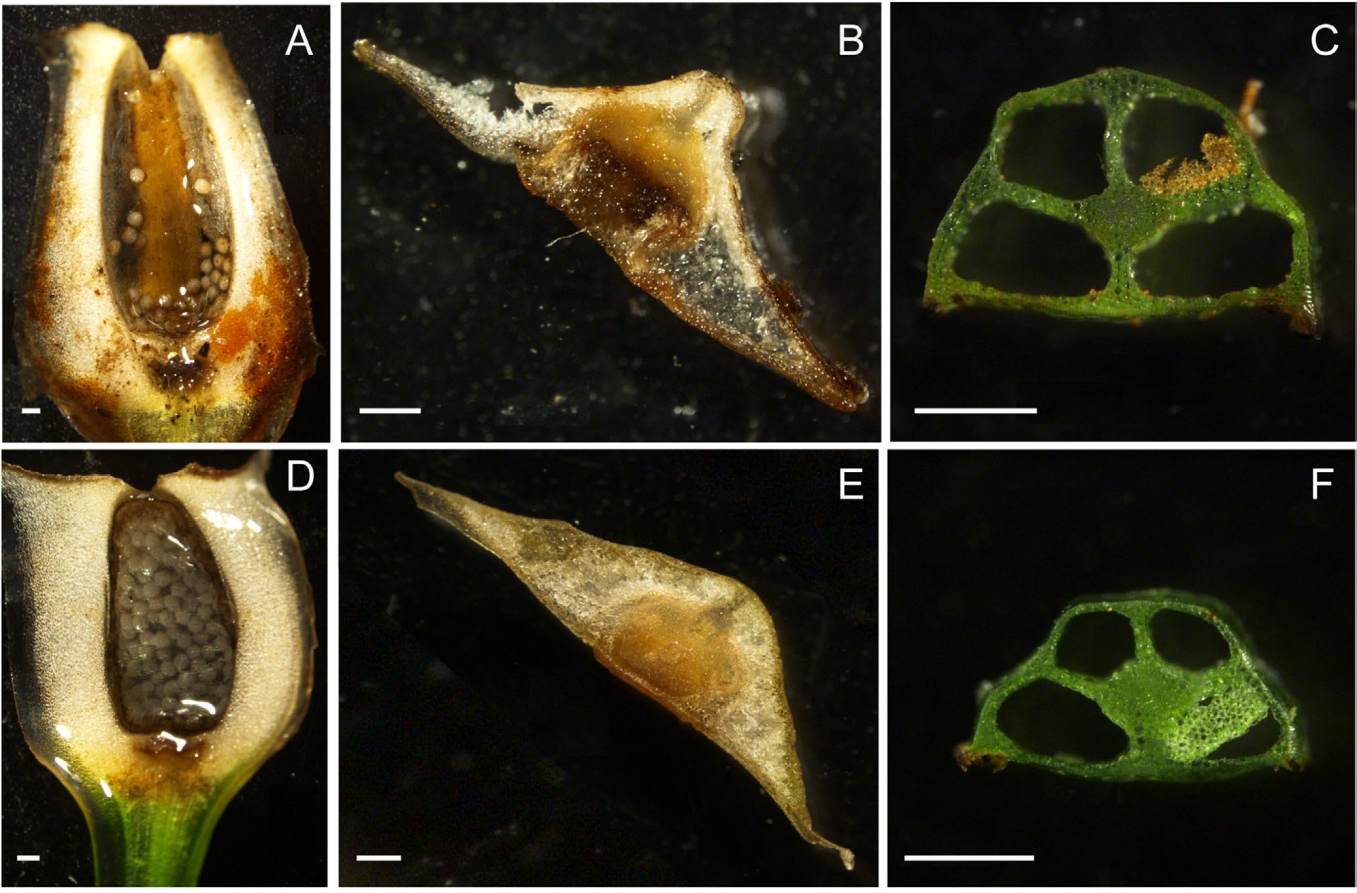
Comparison of *Isoetes fokiensis* and *Isoetes orientalis* regarding Sporangium (A, D), leaf base (just above the sporangium) (B, E), and leaf middle (where leaf wing begin to disappear) (C, F). Scale bar = 200 μm.

**FIGURE 3:**
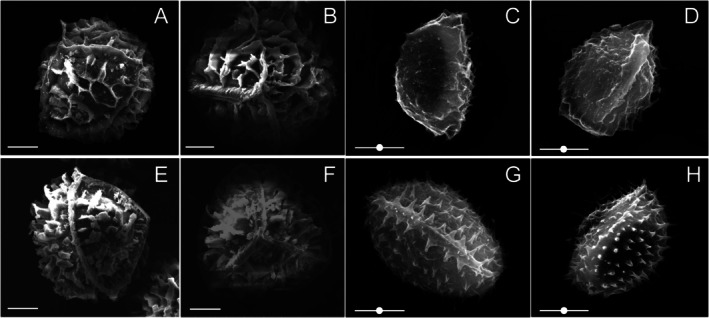
Comparison of *Isoetes fokiensis* (A–D) and *Isoetes orientalis* (E–H) regarding diagram of spore morphology characteristic. (A, B, E, F) Megaspore. (C, D, G, H) Microspore. Scale bar = 100 μm (megaspore) and 10 μm (microspore, with white point).

*Isoetes fokiensis* is most similar to *I. orientalis* , but it differs from the latter by porangium, leaf base, leaf middle, and spore morphology characteristics (Table 2; Figures 2 and 3). The sporangia of *I. fokiensis* are slightly larger and more ellipsoidal, whereas those of *I. orientalis* are smaller and more ovate (Figure 2A,D). Leaves of *I. fokiensis* have conspicuous abaxial ridges, particularly at the base, whereas those of *I. orientalis* are flatter (Figure 2B,E). This difference is also evident in transverse sections of the leaf mid‐region, where *I. fokiensis* shows a more pentagonal outline, in contrast to the trapezoidal shape observed in *I. orientalis* (Figure 2C,F). The megaspores of *I. fokiensis* exhibit relatively high lamellar projections on both the proximal and distal surfaces, forming a reticulate pattern, with a diameter ranging from 450 to 490 μm (mean: 470 μm) (Figure 3A,B). The microspores have a smooth surface or bear sparse granular projections, exhibiting a levigate‐granulate texture, with a diameter ranging from 24 to 27 μm (mean: 25 μm) (Figure 3C,D). Both megaspore and microspore ornamentation of *I. orientalis* are different from *I. fokiensis* (Figure 3E–H; Table 2).

**TABLE 2:** Comparison of altitude, morphological characters between *Isoetes fokiensis* and *Isoetes orientalis*.

Altitude	*I. fokiensis*	*I. orientalis*
	About 1400 m	About 1000 m
Characteristic of leaf base transverse section	Trapezoid, flat ventral surface, distinct ridge	Arched, flat ventral surface, rounded back, inconspicuous ridge
Characteristic of leaf middle transverse section	Slightly pentagonal, flat belly, obscure dorsal ridge	Trapezoidal, flat belly obscure dorsal ridge
Morphology of sporangium	Larger, long elliptic	Smaller, long ovate
Megaspore ornamentation	Reticulate	Cristate‐reticulate
Megaspore size	450–490 μm (mean = 470 μm)	350–460 μm (mean = 420 μm)
Microspore ornamentation	Levigate‐granulate	eChinate‐tuberculate
Microspore size	24–27 μm (mean = 25 μm)	20–38 μm (mean = 34 μm)

### 3.2 Population Genetic Diversity

**FIGURE 4:**
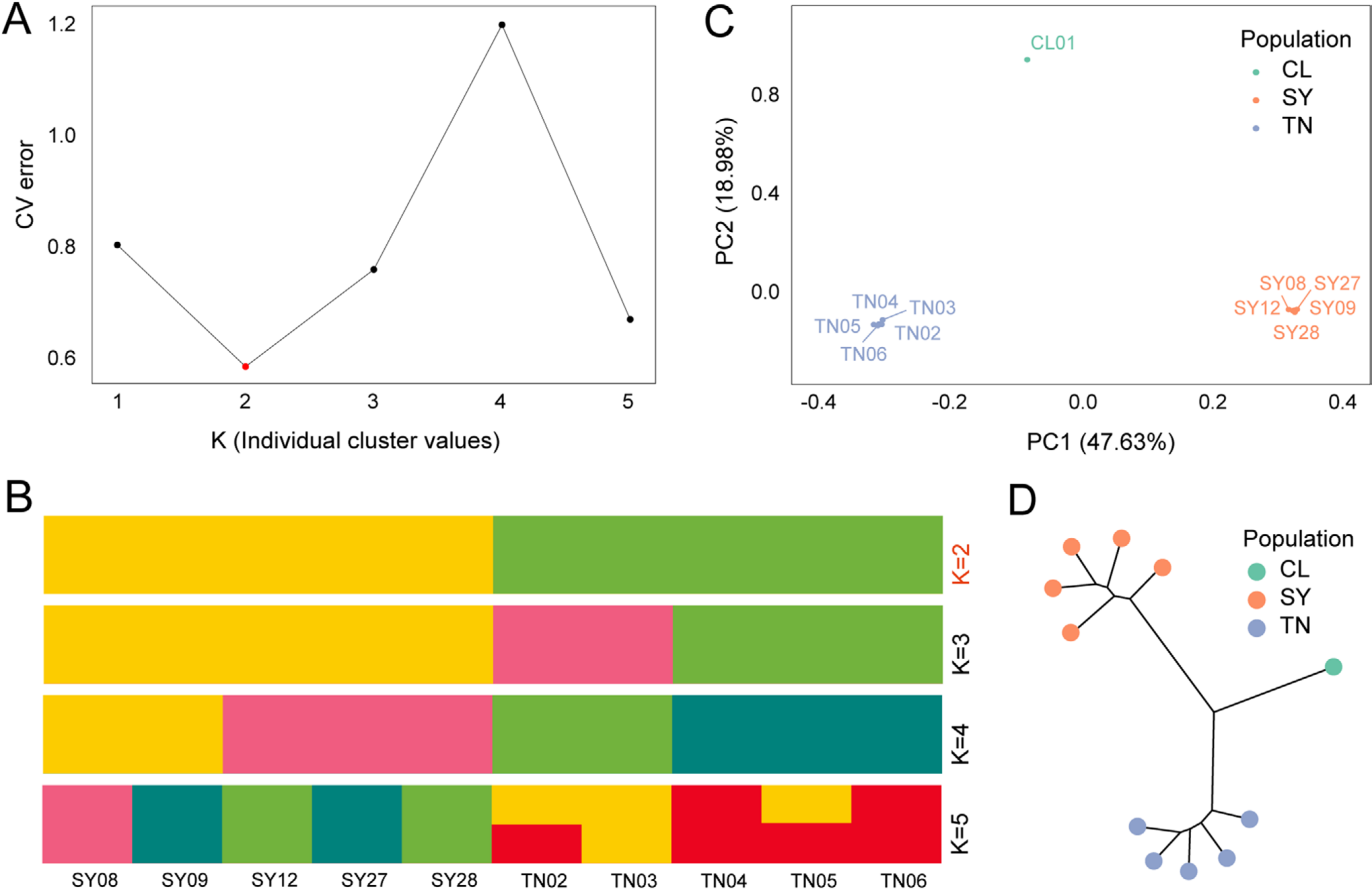
(A) The ADMIXTURE cross‐validation error corresponding to different *K* values. Structure analysis of 10 *Isoetes* individuals at varying *K* values. (B) When *K* = 2, individual clustering was achieved. (C) Principal component analysis (PCA) of 10 *Isoetes* individuals. The inverse of PC1 is plotted on the *x*‐axis, and PC2 is plotted on the *y*‐axis. Colored circles around clusters correspond to the color scheme in Figure 4D and denote the major ancestry of those individuals in STRUCTURE analyses for *K* = 2. (D) Phylogenetic tree of 10 samples based on 94,982 SNPs. CL: *I.changleensis* from Changle Forest farm, Zhejiang. SY: *I.orientalis* from Songyang, Zhejiang. TN: *I.fokiensis* from Taining, Fujian.

A total of 94,982 SNPs were obtained from the CDS‐region genomic data for genetic analysis. Based on the clustering results, the number of clusters with the lowest cross‐validation error rate (CV‐error) was defined as the optimal clustering (Figure 4A). The results showed that when *K* = 2, the CV‐error value was minimized, indicating significant genetic differentiation among the samples and a distant phylogenetic relationship. The results from the ADMIXTURE analysis (Figure 4B) revealed that the samples from different locations represent independent genetic structures, with no gene flow between the distinct populations. This distinction is further corroborated by principal component analysis (Figure 4C).

A comparison of our phylogenetic tree, which is based on the nuclear genome, with previous phylogenetic trees, which are based on the chloroplast genome, revealed consistent results (Gu, Xiang, et al. 2023; Liu et al. 2024). This finding supports the hypothesis that *I. fokiensis* is the sister clade of *I. orientalis* . Outgroup CL01 (*I. changleensis*) also formed an independent clade (Figure 4D). The phylogenetic tree reveals that the intraspecific genetic distances within *I. fokiensis* and *I. orientalis* are considerably lower than the inter‐population distances among the three populations (Figure 4D).

Based on morphological and molecular genetic analyses, we propose that *I. fokiensis* and *I. orientalis* should be recognized as separate species, warranting the designation of *I. fokiensis* as a new species.

## 4 Taxonomic Treatment

**FIGURE 5:**
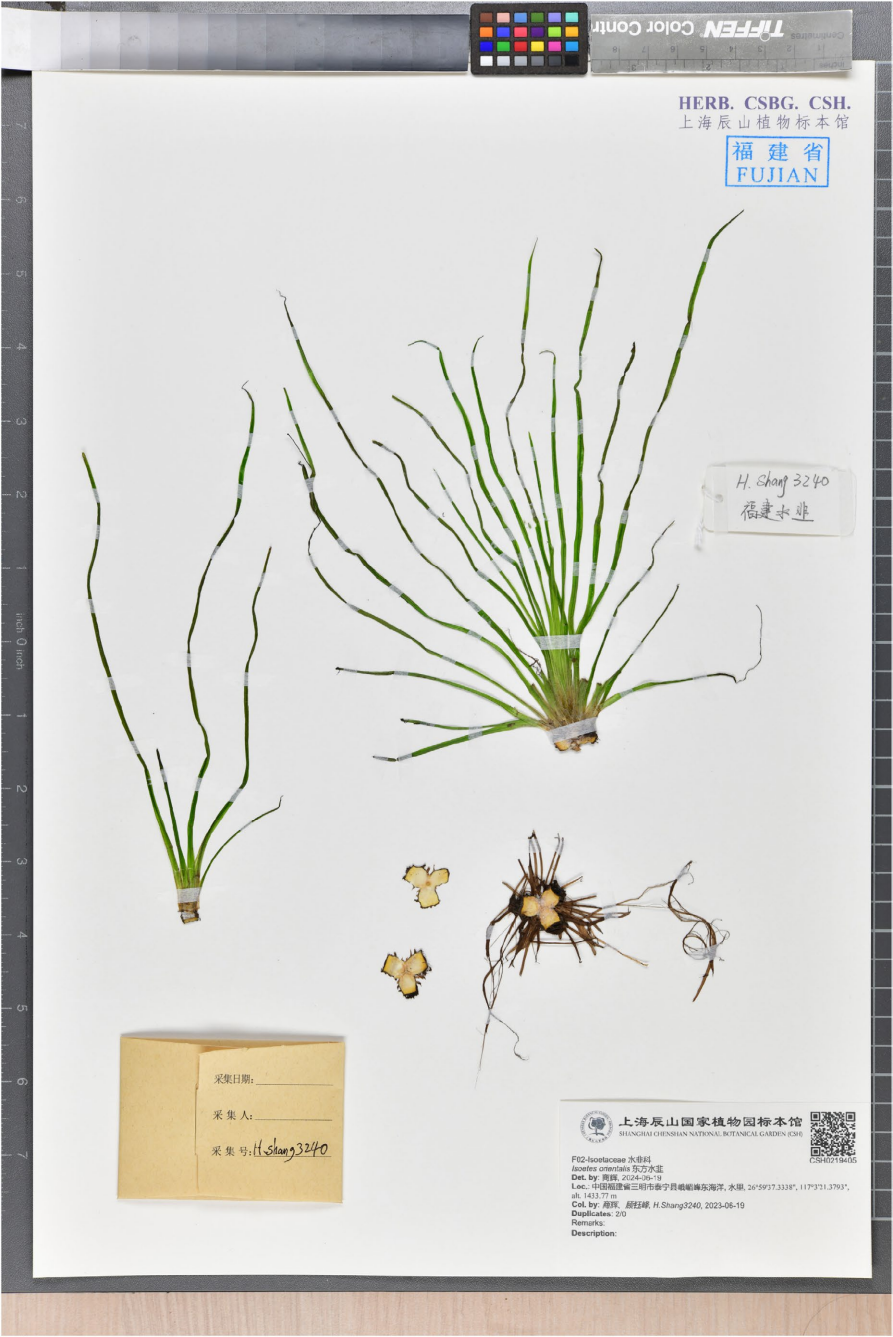
Holotype of *Isoetes fokiensis*.

*Isoetes fokiensis* Y.F.Gu, M.R. Wang & Y.H.Yan, sp. *nov*. (Figures 1, 2A–C, 3A–D, and 5).

福建水韭 fú jiàn shuǐ jiǔ

**Diagnosis:**—Morphologically, *Isoetes fokiensis* is similar to *I. orientalis*. However, *I. fokiensis* differs from *I. orientalis* by its reticulate megaspores on ornamentation characteristics (vs. cristate‐reticulate megaspores), levigate‐granulate microspores (vs. echinate microspores) (Figure 3A–D vs. Figure 3E–H).

**Type:**—CHINA, Fujian Province, Sanming City, Taining Country, Emeifeng National Nature Reserve, elevation 1433.77 m, 26°59′37.3338″N;117°03′21.3793″E, June 19, 2023, *H. Shang and Y.F. Gu, H. Shang3240* (holotype: CSH0219405! isotype: CSH0198211!) (Figure 5).

**Description:**—Plants perennial, semi‐submerged, occurring in alpine wetlands at ~1400 m altitude (Figure 1A,B). Microphylls tufted, several to many, erect to spreading, straight to recurved, fleshy, simple, linear, distichously arranged (Figure 1C), 20–30 cm long, ~1.5 mm wide at the middle; four lacuna separated by transverse septa (Figures 1D and 2C); base white, with wing‐like structures (Figure 2B), dilated and spatulate, tapering to apex, with margins entire and glabrous, lacking serration (Figure 1H). Sporangia, basal, ovate, 7.2 × 3.0 mm, covered by a membranous velum, which is lost at maturity (Figure 2A). Corm tuberous, three lobed (Figure 1E). Megaspores granular, tetrahedral‐spherical, trilete, white when dry, 450–490 μm (mean = 470 μm) in diameter, surface reticulate (Figure 3A,B). Microspores powdery, ellipsoidal, monolete, gray when dry, 24–27 μm (mean = 25 μm) long, surface smooth to granular (Figure 3C,D). Chromosome number: 2n = 6× = 66 (Figure 1F,G).

**Etymology:**—The specific designation comes from the fact that the plant's natural growing place in the wild is in Fujian, China.

**Distribution and Habitat:**—*Isoetes fokiensis* is only known from one locality, Fujian Emeifeng National Nature Reserve (Figure 1B). The plants grow in the wetlands of mountain streams at an altitude of about 1200 m (Figure 1C), and its sporulation period is from June to November. This region has a mid‐subtropical, marine monsoon‐type mountainous climate, with additional continental climatic characteristics. The mean annual temperature is 15.4°C, with an average of 4.4°C in the coldest month (January) and 25.3°C in the warmest month (July). The average frost‐free period is 286 days per year, the mean annual precipitation is 1913 mm, and the average annual relative humidity exceeds 82%.

**Paratype:**—CHINA, Fujian Province, Sanming City, Taining Country, Emeifeng National Nature Reserve, elevation 1433.77 m, 26°59′37.3338″N;117°03′21.3793″E, 31 August 2019, *Y.F.Gu, Fern08747*, NOCC!

## 5 IUCN Red List Category

*Isoetes fokiensis* sp.nov. is currently found in only one location in Emeifeng National Nature Reserve, Taining Country, Fujian Province, China, with two populations (128 individuals in total, occupying an area of < 10 km^2^). Thus, this species should be considered as critically endangered (CR) [B2ab(iii); C2a(i)] according to the Red List Categories and Criteria (IUCN 2024) due to its current narrow geographical distribution, especially the shrinkage of its wetland habitat caused by human activities and climate change.

## 6 Key to *Isoetes* of China

1. Leaves 1‐2 mm wide at middle‐‐‐‐‐‐‐‐‐‐‐‐‐‐‐‐‐‐‐‐‐‐‐‐‐‐‐‐‐‐‐‐‐‐‐‐‐2

‐. Leaves 2‐10 mm wide at middle‐‐‐‐‐‐‐‐‐‐‐‐‐‐‐‐‐‐‐‐‐‐‐‐‐‐‐‐‐‐‐‐‐‐‐‐9

2. Small ferns, mostly found at high altitudes (H > 2000 m)‐‐‐‐‐‐‐‐‐‐‐‐‐‐‐‐‐‐‐‐‐‐‐‐‐‐‐‐‐‐‐‐‐‐‐‐‐‐‐‐‐‐‐‐‐‐‐‐‐‐‐‐‐‐‐‐‐‐‐‐‐‐3

‐. Taller plants, mostly found at low to medium altitudes (H < 2000 m)‐‐‐‐‐‐‐‐‐‐‐‐‐‐‐‐‐‐‐‐‐‐‐‐‐‐‐‐‐‐‐‐‐‐‐‐‐‐‐‐‐‐‐‐‐‐‐‐‐‐‐‐‐‐‐‐‐‐‐‐‐4

3. Megaspore laevigate, microspore regulate‐‐‐‐‐‐‐‐‐‐‐‐‐‐‐‐‐‐‐‐‐‐‐‐‐‐‐‐‐‐‐‐‐‐‐‐‐‐‐‐‐‐‐‐‐‐‐‐‐‐‐‐‐‐‐‐‐‐‐‐‐‐‐‐‐‐‐‐‐‐‐‐‐‐‐‐‐‐‐ *Isoetes hypsophila*

‐. Megaspore tuberculate‐rugulate, microspore echinate to cristate‐‐‐‐‐‐‐‐‐‐‐‐‐‐‐‐‐‐‐‐‐‐‐‐‐‐‐‐‐‐‐‐‐‐‐‐‐‐‐‐‐‐‐‐‐‐‐‐‐*I. shangrilaensis*

4. Megaspore cristate‐reticulate, microspore echinate‐tuberculate‐‐‐‐‐‐‐‐‐‐‐‐‐‐‐‐‐‐‐‐‐‐‐‐‐‐‐‐‐‐‐‐‐‐‐‐‐‐‐‐‐‐‐‐‐‐‐‐‐‐*I. orientalis*

‐. Megaspore cristate, regulate, tuberculate or tuberculate‐cristate, microspore echinate‐‐‐‐‐ ‐‐‐‐‐‐‐‐‐‐‐‐‐‐‐‐‐‐‐‐‐‐‐‐‐‐‐‐‐‐‐‐‐‐‐‐‐‐‐‐‐‐‐‐‐‐‐‐‐‐5

5. Individual diploid (2n = 22)‐‐‐‐‐‐‐‐‐‐‐‐‐‐‐‐‐‐‐‐‐‐‐‐‐‐‐‐‐‐‐‐‐‐‐‐‐‐‐‐‐6

‐. Individual tetraploid (2n = 44)‐‐‐‐‐‐‐‐‐‐‐‐‐‐‐‐‐‐‐‐‐‐‐‐‐‐‐‐‐‐‐‐‐‐‐‐‐‐8

6. Just distributing in Taiwan‐‐‐‐‐‐‐‐‐‐‐‐‐‐‐‐‐‐‐‐‐‐‐‐‐‐‐*I. taiwanensis*

‐. Distributing in Zhejiang‐‐‐‐‐‐‐‐‐‐‐‐‐‐‐‐‐‐‐‐‐‐‐‐‐‐‐‐‐‐‐‐‐‐‐‐‐‐‐‐‐‐‐‐‐‐7

7. Megaspore ornamentation tuberculate‐‐‐‐‐‐‐‐‐‐‐*I. changleensis*

‐. Megaspore ornamentation regulate‐‐‐‐‐‐‐‐‐‐‐‐‐‐‐‐*I. yuhangensis*

8. Megaspore cristate, 340–450 μm (mean = 409 μm) in diameter on the proximal face‐‐‐‐‐‐‐‐‐‐‐‐‐‐‐‐‐‐‐‐‐‐‐‐‐‐‐‐‐‐‐‐‐‐‐‐‐‐‐‐‐*I. sinensis*

‐. Megaspore tuberculate‐cristate, 280‐410 μm in diameter on the proximal face‐‐‐‐‐‐‐‐‐‐‐‐‐‐‐‐‐‐‐‐‐‐‐‐‐‐‐‐‐‐‐‐‐‐‐‐‐‐‐‐‐*‐I. longpingii*

9. Megaspore reticulate or cristate‐reticulate‐‐‐‐‐‐‐‐‐‐‐‐‐‐‐‐‐‐‐‐‐‐10

‐. Megaspore echinate‐cristate‐‐‐‐‐‐‐‐‐‐‐‐‐‐‐‐‐‐‐‐‐‐‐‐‐‐‐‐‐‐‐‐‐‐‐‐‐‐‐12

10. Individual diploid (2n = 22)‐‐‐‐‐‐‐‐‐‐‐‐‐‐‐‐‐‐‐‐‐‐‐‐*I. yunguiensis*

‐. Individual hexaploid (2n = 66)‐‐‐‐‐‐‐‐‐‐‐‐‐‐‐‐‐‐‐‐‐‐‐‐‐‐‐‐‐‐‐‐‐‐‐‐‐11

11. Microspore echinate‐‐‐‐‐‐‐‐‐‐‐‐‐‐‐‐‐‐‐‐‐‐‐‐‐‐‐‐‐‐‐‐‐‐‐‐‐‐‐‐*I. fengii*

‐. Microspore levigate‐granulate‐‐‐‐‐‐‐‐‐‐‐‐‐‐‐‐‐‐‐‐‐‐‐‐‐‐*I. fokiensis*

12. Individual diploid (2n = 22)‐‐‐‐‐‐‐‐‐‐‐‐‐‐‐‐‐‐‐‐‐‐‐‐‐‐*I. baodongii*

‐. Individual tetraploid (2n = 44)‐‐‐‐‐‐‐‐‐‐‐‐‐‐‐‐‐‐‐‐‐‐‐‐‐‐‐‐‐‐‐‐‐‐‐‐‐13

13. Megaspore 390–450 μm (mean = 430 μm) in diameter on the proximal face ‐‐‐‐‐‐‐‐‐‐‐‐‐‐‐‐‐‐‐‐‐‐‐‐‐‐‐‐‐‐‐‐‐‐‐‐‐‐‐‐‐‐‐‐‐‐‐‐‐*I. xiangfei*

‐. Megaspore 317–411 μm (mean = 360 μm) in diameter on the proximal face‐‐‐‐‐‐‐‐‐‐‐‐‐‐‐‐‐‐‐‐‐‐‐‐‐‐‐‐‐‐‐‐‐‐‐‐‐‐‐‐‐*I. changxingensis*

## Author Contributions

**Moran Wang:** conceptualization (supporting), data curation (lead), formal analysis (lead), investigation (equal), methodology (lead), project administration (supporting), software (lead), validation (equal), visualization (lead), writing – original draft (lead), writing – review and editing (lead). **Hui Shang:** conceptualization (equal), investigation (equal), supervision (supporting), validation (equal), writing – review and editing (equal). **Wen Shao:** investigation (equal), validation (equal), visualization (supporting). **Binjie Ge:** investigation (equal), validation (equal), visualization (supporting). **Yigang Song:** resources (equal). **Yuehong Yan:** resources (equal), supervision (equal). **Weimin Ma:** resources (equal), supervision (supporting). **Yufeng Gu:** conceptualization (lead), funding acquisition (equal), investigation (equal), supervision (supporting), validation (equal), visualization (equal), writing – review and editing (equal). **Hui Shen:** conceptualization (equal), funding acquisition (equal), investigation (equal), project administration (lead), resources (lead), supervision (lead), writing – review and editing (equal).

## Conflicts of Interest

The authors declare no conflicts of interest.

## Data Availability

The variation data reported in this paper have been deposited in the Genome Variation Map (GVM) in the National Genomics Data Center, Beijing Institute of Genomics, Chinese Academy of Sciences and China National Center for Bioinformation, under accession number GVM001065 (https://bigd.big.ac.cn/gvm/getProjectDetail?Project=GVM001065); Genome Variation Map: a worldwide collection of genome variations across multiple species. Nucleic Acids Res 2021, 49(D1):D1186‐D1191 (PMID = 33170268). Database Resources of the National Genomics Data Center, China National Center for Bioinformation in 2022, Nucleic Acids Res 2022 (PMID = 34718731).
