# Correction to “A New Hexaploid Species of *Isoetes* (Isoetaceae) From Fujian, China, Based on Morphological and Molecular Evidence”

**DOI:** 10.1002/ece3.74187

**Published:** 2026-08-24

Wang, M. , H. Shang , W. Shao , et al. 2025. “A New Hexaploid Species of *Isoetes* (Isoetaceae) From Fujian, China, Based on Morphological and Molecular Evidence.” Ecology and Evolution 15, no. 9: e72140. 10.1002/ece3.72140.40950794 PMC12426898

In Figure 3G, the original source of the image was not cited. The image was previously published in Li et al. (2023). Accordingly, the caption of Figure 3 should be updated, and the following reference should be added to the References.

Li, Y., X. Chen, X. Lin, Y. Gu, B. Liu, B, and R. Zhang. 2023. “Complete Chloroplast Genome of *Isoetes orientalis* (Isoetaceae), an Endangered Quillwort From China.” *Mitochondrial DNA Part B* 8, no. (3): 342–346. https://doi.org/10.1080/23802359.2023.2183070.

Figure 3 and its corrected caption are provided below.

**FIGURE 3:**
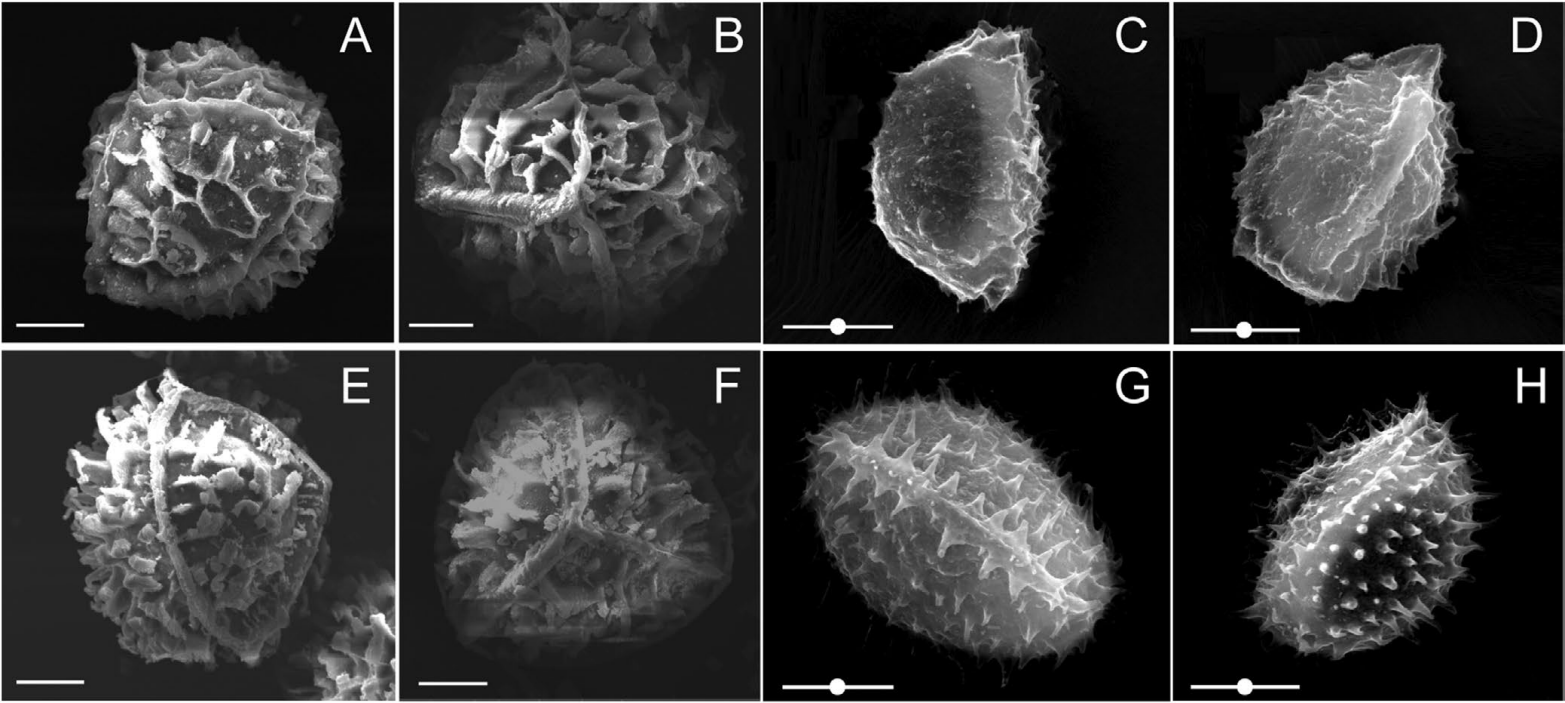
Comparison of *Isoetes fokiensis* (A–D) and *Isoetes orientalis* (E–H) regarding diagram of spore morphology characteristic. (A, B, E, F) Megaspore. (C, D, G, H) Microspore. Scale bar = 100 μm (megaspore) and 10 μm (microspore, with white point). (G) Image adapted from Li et al. 2023, with independent data and analysis.

We apologize for this error.

